# Investigating the Impact of the AI-Supported 5E (AI-s5E) Instructional Model on Spatial Ability

**DOI:** 10.3390/bs14080682

**Published:** 2024-08-06

**Authors:** Nejla Gürefe, Gülfem Sarpkaya Aktaş, Hava Öksüz

**Affiliations:** 1 Faculty of Education, Mersin University, Mersin 33100, Turkey; 2 Faculty of Education, Çukurova University, Adana 01330, Turkey; gsarpkaya@cu.edu.tr (G.S.A.); 2022933157@ogr.cu.edu.tr (H.Ö.)

**Keywords:** spatial ability (SA), artificial intelligence (AI), 5E instructional

## Abstract

Improving students’ spatial abilities is an important goal in education. Spatial ability is a skill needed in many fields, such as science, mathematics, engineering, and architecture. Since this ability can be improved through training, this study adopted a quasi-experimental design to investigate the effects of an artificial intelligence-supported 5E (AI-s5E) instructional model on students’ spatial visualization, spatial relationships, and spatial orientation performances that explain their spatial abilities. A total of 43 students from two classes at a secondary school in western Turkey were recruited to participate in this study. One of the classes was the experimental group (f = 23), which adopted the AI-s5E approach, and the other class was the control group (f = 20), which adopted the traditional teaching model. The results showed that the integration of the AI-s5E instructional approach into education improved students’ spatial abilities and sub-dimensions. In light of the findings, it can be recommended that AI applications, which have a positive and significant impact on spatial skills, can be integrated into teachers’ lessons and even included in curriculum programs.

## 1. Introduction

The adoption and integration of new technologies in the classroom can raise the standard of mathematics education and promote equity [1]. Therefore, there is a need for quality computer education that will enable students to explore their abilities and develop their cognitive thinking [2]. One of these technologies is artificial intelligence (AI). AI describes a set of advanced general-purpose digital technologies that enable machines to perform highly complex tasks effectively [2]. The integration of AI into the education process is very important, both for closely following the technology and for future generations [3]. The lack of educational perspectives in AI education (AIEd) research, development, or applications is constantly mentioned [4,5,6,7]. Therefore, one of the most critical steps to further develop AI technologies in education is to fully involve educators and educational researchers in the technological innovation process. Actively seeking feedback from educational communities and integrating theoretical, conceptual, practical, and empirical knowledge from the educational literature is an important part of this process [8]. According to Ee and Huh [9], students will live in an educational environment where AI tools are used with the developing technology. As a matter of fact, with the use of AI in education, better development of educational environments will be ensured [10]. The progress of AIEd will also be realized as a result of more empirical studies focusing on AI technologies in real teaching and learning environments that serve educational needs and goals [6]. It is important to implement learning environments where teaching methods are enriched using AI technology and to evaluate them in terms of various factors.

One of the potential benefits of AI technologies for education is visualization and a visual learning environment [8]. Although visualization is encountered in all areas of mathematics, it is more common in geometry teaching. In geometry, defining the properties and relationships of shapes, angles, and lines [11] and representing geometric problems with shapes are generally at the forefront. Therefore, these are closely related to visualization processes [12]. SA, which is the ability to create mental representations of the visual stimuli we see around us and to manipulate these images in the mind, plays an important role in overcoming many tasks in daily life and especially in various disciplines such as mathematics and geometry and has a positive relationship [13]. The ability to define objects and situations in the mind and manipulate the images formed is an important cognitive skill for most career fields, especially graphic and geometric drawing [14].

SA is defined as the ability to visually manipulate, reorganize, or interpret relationships [15]. There are many studies in the literature indicating that the components of SA are “spatial visualization”, “spatial relations”, and “spatial orientation” [16,17,18]. This study was conducted by taking the mentioned theoretical framework into consideration. In this case, the definition of spatial relations is considered as the ability of the learner to translate two- and three-dimensional geometric forms as a whole in his/her mind and to recognize them in various positions [19], the definition of spatial orientation as the ability to understand and use the relationships between different positions in space according to one’s own position [20], and the definition of spatial visualization as the ability to manipulate imaginary movements or objects in imagination in a three-dimensional space [17].

There are studies linking SA with mathematical ability [21,22]. For example, Cheng and Mix [22] tested whether mental rotation training improves math performance in 6- to 8-year-old children. They found that children improved significantly in calculation problems thanks to the training they received in the spatial training group. In another study, Xie et al. [12] investigated the relationship between spatial and mathematical ability. Their meta-analysis showed that the relationship between mathematics and SA is not simply linear. The relationship between logical reasoning and SA was found to be stronger than the relationship between numerical or arithmetic ability and SA. They also showed that geometric ability has a strong relationship with SA. Geometric ability is defined as understanding the motion, transformation, and spatial relationships of planes and solids and the geometric interpretation of mathematical and algebraic formulas [12]. Learning geometry, especially 3D geometry, requires SA, especially the representation of 3D objects in a 2D view [14]. Cross-sectional images of objects are difficult to learn by students who do not have strong basic knowledge about the object. Moreover, there are several concepts in geometry that require students to define objects and identify their properties by distinguishing them from existing experience. This concept of geometry also requires visual interpretation of geometric problems presented in 2D in question papers. If students cannot extract 3D geometry information drawn in isometric view on paper, they may have difficulty interpreting questions involving 3D geometry [14]. For this reason, SA is important for students both in their educational life and in their later life.

When the studies on SA are examined, the SA [23,24] and its subcomponents, spatial visualization [25,26,27,28], mental rotation [29], and spatial relations [28], skills of middle school students were determined, and different variables affecting these levels were revealed. On the other hand, in some studies on SA, geometric–mechanical intelligence games [30,31], computer software [32], digital games [33,34,35,36,37], manipulatives [38], robots [39,40,41,42], interactive whiteboards [43], android learning media [44], and augmented reality [45]. There is no study on the development of SA in learning environments designed with the AI-s5E instructional model. Therefore, it is clear that the effects of AI-supported activities discussed in the current study will meet the need for pedagogical support in teaching and assessing SA. Therefore, more studies are needed to reveal the effectiveness of AI-supported activities and the teaching process. In addition, it is thought that the designed activities can be an example for pre-service teachers to use AI in the teaching environment. Therefore, it is important to plan and conduct studies to reveal the effectiveness of AI-supported activities and the teaching process. As a matter of fact, it is thought that studies on the development of individuals who use 3D geometry effectively with AI technologies to support their SA, especially studies with students who transition from concrete to abstract, will contribute to the literature. Therefore, the aim of this study is to propose a learning environment enriched with AI-supported tools to improve the SA of middle school students. The following research questions were addressed to evaluate the effectiveness of the proposed learning environment:

1. Do the AI-s5E instructional models show a significant difference in students’ Spatial Ability Test (SAT) scores?
2. Do the AI-s5E instructional models show a significant difference in students’ Spatial Visualization Test (SVT) scores?
3. Do the AI-s5E instructional models show a significant difference in students’ Spatial Relations Test (SRT) scores?
4. Do the AI-s5E instructional models show a significant difference in students’ Spatial Orientation Test (SOT) scores?

### 1.1. Literature Review

#### 1.1.1. AI in Education

Integrating AI into the education process is very important in order to follow technology closely and raise future generations [3]. With the help of AI technologies that simulate human intelligence to make inferences, judgments, or predictions, computer systems provide personalized guidance, support, or feedback to students, as well as assisting teachers or policymakers in decision-making [46]. AI helps students find topics they are curious about faster and easier. All course-related information can be easily accessed using AI. In this century, students are more inclined and willing to learn and discover new knowledge on their own, so this aspect of AI can help students discover more without waiting for an educator [47]. Due to these features, there is concern that AI may replace educators and teachers. However, AI will never take over an educator’s job [48]. AIEd technologies can be used as teacher assistants, automating knowledge assessment, analyzing students’ behavior, and offering intelligent, adaptable, or customizable learning systems [3]. The most common AIEd technologies are chatbots, expert systems, intelligent tutors/agents, machine learning, personalized learning systems/environments, and visualization-based virtual learning environments [8]. The advancement of AIEd will also be realized as a result of more empirical studies focusing on AI technologies in real teaching and learning environments [6] that serve educational needs and purposes.

AI applications offer various opportunities to individuals in the teaching and learning process [7,49,50]. As a teacher assistant, automating knowledge assessment, analyzing students’ behaviors, and providing intelligent, adaptive, or customizable learning systems [3] are some of these opportunities. The main goal of the use of AI in education is to explore new teaching and learning methods and to advance the thinking and learning abilities of the 21st century [51]. Furthermore, for students, AIEd can facilitate a variety of interactions, increase student engagement, create adaptive learning materials, offer meta-cognitive cues, provide enriched learning environments, and improve learning outcomes. It can also enrich the learning experience with visualization and immersive technologies in education [8]. In addition, AI tools have the ability to develop and adapt over time with their dynamic and learning algorithms [52]. With this technology, it can be aimed at providing an effective learning experience by providing students with personalized learning advice and support in accordance with their learning needs, preferences, and personal characteristics [53]. In their educational lives, students can benefit from individualized learning opportunities, and teachers can use the latest teaching methods [54]. In this case, it can be said that AI tools have the potential to make the learning experience more interactive and individualized than others. These features actually differentiate it from other technologically supported tools.

Visualization and virtual learning environments, one of the AIEd technologies, can enrich the learning experience with visualization and immersive technologies in education. Due to this feature, it can be said that it can be used in teaching geometry subjects where visualization is at the forefront. Since geometry is mainly expressed as defining the properties of shapes, angles, and lines and the relationships between them [11], shapes and 2D and 3D representations of these shapes are generally prominent in geometric problems. Therefore, these are closely related to visualization processes [12]. Animating and representing shapes and mathematical representations obtained by using appropriate AI-supported software in mathematics education also increases students’ imagination and problem-solving skills [55]. Arıca Oueini [56] investigated the effects of AIEd, an AI web-based software program, on 11th grade students’ geometry achievement, self-efficacy, and attitudes towards mathematics. He found that AIEd had a positive effect on student achievement, but the results were not statistically significant, and AIEd positively affected students’ self-efficacy and attitudes by helping them understand and enjoy mathematics.

In the systematic literature review study on the use of AI in mathematics education, only two of the studies using AI in mathematics education use AI-supported mathematical tools [47]. Among these studies, Dunzhin and Gustafsson [57] evaluated the methods used in the mathematics classroom by developing a machine-learning algorithm that takes into account students’ prior knowledge and revealed that active teamwork is more beneficial for students than individual work. They integrated the algorithm they produced into an application and shared it with the education community. However, for AI to be effectively integrated into mathematics education, certain parameters must be met. These parameters include defining the role of the teacher, establishing appropriate usage limits, and creating environments suitable for teaching methods [55]. When research on AI in education is examined, personalized teacher systems, learning systems, and environments, as well as machine learning technologies, are widely used [8,58]. There are studies on the application of chat robots [59]. Korkmaz Güler et al. [59] chatGPT, one of the chatbots in their study They compared the performance of two different versions of solving mathematical problems. As a result of their studies, they stated that chatGPT 4 was more successful in problem solving than 3.5. Hwang and Tu [58] revealed the relationships between the roles of AI and the educational supports provided by AI in their bibliometric and systematic study of studies investigating AI in mathematics education. They state that in these studies, where the main educational supports provided by smart teaching systems are evaluated in terms of student models and academic success, the most important ones are diagnosing strengths and giving automatic feedback, as well as examining teachers’ perspectives. In addition, they revealed that their studies focused on diagnosing their students’ mathematics learning problems and providing them with feedback to reduce the teachers’ burden. On the other hand, issues related to organizing learning materials for individual students and facilitating collaboration appear to be rarely explored within teaching systems. It seems appropriate to diversify AI-supported learning environments and applications.

#### 1.1.2. Spatial Ability (SA)

The earlier students are introduced to SA, which has a significant impact on daily and professional life, the easier it is to develop and support this skill [26].

While defining SA, researchers have not been able to agree on different definitions for spatial skill and ability, the number of types of SA, different numbers and names given to describe the subcomponents of SA, and the existence of many different tests measuring different subcomponents of SA [60]. For example, Xie et al. [12] defined five areas of SA in their meta-analysis study, where they revealed the relationship between SA and mathematical ability: internal-dynamic (ID), internal-static (IS), external-dynamic (ED), external-static (ES), and visual–spatial memory. Intrinsic-dynamic SA refers to processing objects or shapes by physically or mentally transforming them, as required by block design, mental rotation, and paper folding tasks. External-dynamic SA refers to processing relationships between objects or shapes by physically or mentally transforming them, as required by perspective-taking, spatial navigation, and dynamic map reading tasks. Extrinsic static SA refers to the processing of relationships between objects or shapes without transformation, as required by water level and static map reading tasks. Dere and Kalelioğlu [26] defined the definition of SA in their research. They defined mental manipulation of space and objects in space as ‘the ability to perform mental transformation of objects in space, to visualize how objects appear from different angles, and to understand how objects are related to each other’, using expressions based on concepts such as the movement, transformation, and relationships of objects. SA includes producing, storing, retrieving, and transforming visual information [61]. Based on all these definitions, the components of SA are ‘spatial visualization’, ‘spatial relations’, and ‘spatial orientation’ [16,17,18]. Spatial relations are the student’s ability to move two- and three-dimensional geometric forms in his mind as a whole and recognize them in various positions [19], spatial orientation is the ability to understand and use the relationships between different positions in space according to one’s own position [20], and spatial visualization is defined as the ability to manipulate imaginary movements or objects in imagination in a three-dimensional space [17].

There is research on SA. In addition to measuring SA and its subcomponents, these studies were also conducted on learning environments to develop SA. For example, in their study, Dere and Kalelioğlu [26] investigated the effects of the applications they created using a web-based 3D design environment on the spatial visualization and mental rotation abilities of secondary school students. As a result, they found an increase in the students’ abilities. Another study investigated the potential benefit of educational robotics in improving SA, and as a result, it was found that the positive change in the spatial abilities of those who participated in the robotics course was significantly greater than the change in students who did not participate in the course [40]. As stated in the introduction of the article, the effects of geometric mechanical intelligence games, manipulatives, interactive whiteboards, and digital games on SA have been the subject of research. The purpose of this research is the change in the spatial abilities of secondary school students who use applications supported by AI from new technologies.

## 2. Materials and Methods

### 2.1. Research Design

**Figure 1:**
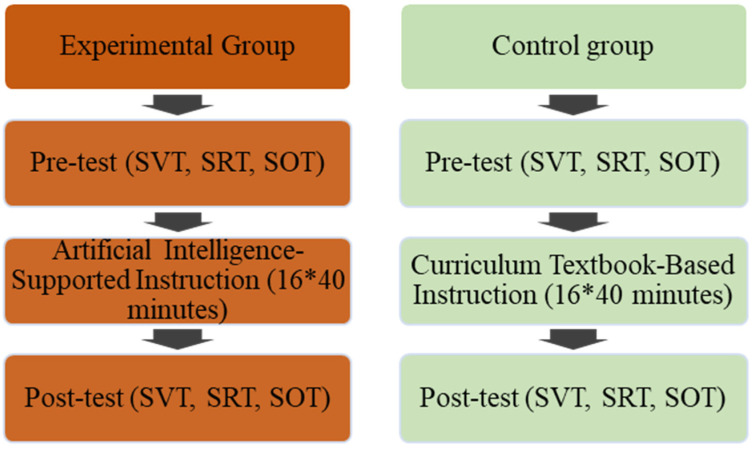
Experimental design of this study. SVT: spatial visualization test; SRT: spatial relations test; SOT: spatial orientation test.

In this study, a quasi-experimental design consisting of a pre-test and post-test was used. Quasi-experimental designs are preferred in cases where it is not possible to form the classes through random assignment to experimental and control groups by the researchers [62]. Two fifth-grade classes were selected for our study, and these classes are educated in the same state secondary school. One of the selected classes was determined as the experimental group and the other as the control group. With the students in the experimental group, the AI-s5E teaching model was applied. The students in the control group were taught in accordance with the course book, and no intervention was made. Our study was carried out in the spring term of the 2024 academic year. One of the researchers in this study is a mathematics teacher in a public school and has experience with AI-supported tools. This researcher carried out the teaching with the experimental and control groups in this study. At the beginning of this study, a Spatial Visualization Test (SVT), a Spatial Relations Test (SRT), and a Spatial Orientation Test (SOT) were applied to the experimental and control groups as a pre-test. In addition, a Spatial Visualization Test (SVT), a Spatial Relations Test (SRT), and a Spatial Orientation Test (SOT) were administered to both groups at the end of the instruction. The research design is provided in Figure 1.

### 2.2. Study Group

The participants of this study were 43 secondary school students studying in a central public school in the district of a city located in the western region of Turkey. The participants were divided into two groups: 23 (13 males and 10 females) in the experimental group (EG) and 20 (12 males and 8 females) in the control group (CG). The participants were between the ages of 10 and 12, and the average age of the participants was 11.69. It was determined that the socio-economic status of the students in both groups was similar, and the groups were randomly assigned as EG and CG. In addition, the reason for choosing this public secondary school in this study was that the students in the experimental group had tablets or computers and sufficient equipment in terms of internet. Besides this, school administration provided voluntary participation for this study to be conducted in their schools. All students participating in this study had previously taken courses that improved their computer literacy. The parents of the participants were informed about the use of AI technology during this study, and the necessary permissions were obtained.

### 2.3. Data Collection Tools

The Spatial Visualization Test (SVT), Spatial Relation Test (SRT), and Spatial Orientation Test (SOT) measure the skills of the subcomponents of SA and were used as data collection tools in this study. The Spatial Visualization Test (SVT), Spatial Relations Test (SRT), and Spatial Orientation Test (SOT) were modified from Dokumacı Sütçü [13].

The Spatial Visualization Test (SVT) consists of 29 items and has a two-factor structure. The first factor consists of the first 14 items measuring 2D spatial visualization skills, and the second factor consists of the last 15 items measuring 3D spatial visualization skills. The 2D spatial visualization questions in the first factor consist of mental integration, mental decomposition, and paper folding skills. The 3D spatial visualization questions in the second factor consist of mental decomposition, number of cube contacts, cube counting, and cube unfolding skills. In the test, 1 point was given for correct answers, and 0 points were given for incorrect answers or answers left blank. The maximum score that can be obtained from the test for the first factor part is 14, and the minimum score is 0. The maximum score that can be obtained for the second factor part is 15, and the minimum score is 0. The maximum score that can be obtained from the overall test is 29, and the minimum score is 0. The KR-20 internal consistency coefficient for the first factor part of the test was calculated as 0.77, and the KR-20 internal consistency coefficient for the second factor part of the test was calculated as 0.78. The overall internal consistency coefficient of the test was calculated as 0.78.

The Spatial Relations Test (SRT) consists of 21 items and has a two-factor structure. The first factor consists of the first 10 items measuring 2-dimensional spatial association skills, and the second factor consists of the last 11 items measuring 3-dimensional spatial association skills. In the test, 1 point was given for correct answers, and 0 points were given for incorrect answers or answers left blank. The maximum score that can be obtained from the test for the first factor part is 10, and the minimum score is 0. The maximum score that can be obtained for the second factor part is 11, and the minimum score is 0. The maximum score that can be obtained from the overall test is 21, and the minimum score is 0. The KR-20 internal consistency coefficient for the first factor part of the test was calculated as 0.79, and the KR-20 internal consistency coefficient for the second factor part of the test was calculated as 0.73. The overall internal consistency coefficient of the test was calculated as 0.74.

The Spatial Orientation Test (SOT) consists of 10 items and has a single-factor structure. The test consists of questions measuring the view of objects from different directions and unit cube number skills. The maximum score that can be obtained from the test is 10, and the minimum score is 0. The KR-20 internal consistency coefficient of the test was calculated as 0.71.

### 2.4. Instruction Process in Experiment and Control Groups

The teaching in both groups was carried out by the teacher of the researchers. At the time of this study, the mathematics teacher had a master’s level education and knowledge and experience in the use of AI-supported tools. The instruction in the groups started in the same week and continued for a total of 16 lesson sessions, each lasting 40 min. While the teaching in the experimental group was carried out according to the 5E instructional process involving AI-supported tools, the teaching in the control group continued with activities based on the curriculum textbook. The lesson plans used in the experimental group were asked to be examined by a mathematics teacher and two mathematics experts involved in the research. After the teacher and expert opinions, the plans were revised and started to be implemented.

#### 2.4.1. Experimental Group

**Figure 2:**
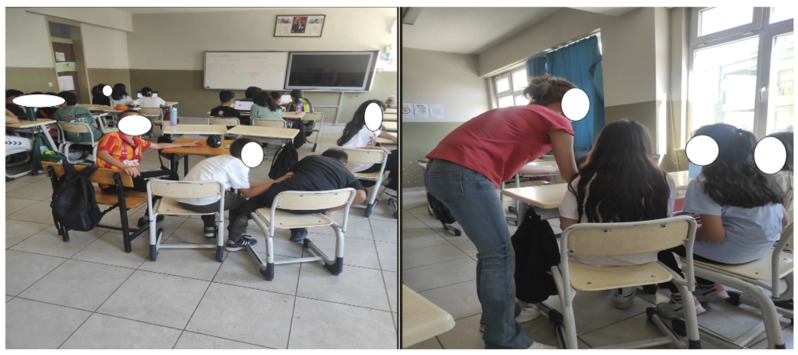
Example of classroom environment.

The experimental group studies were carried out in the classroom environment since there was no computer class in the school. The students were divided into groups (7 groups of 3 students and one group of 2 students). Each group had a tablet or laptop in front of them. The teaching process in the experimental group was carried out with an interactive whiteboard and tablet or laptop (see Figure 2).

In the experimental group, the teacher conducted the teaching process according to the 5E instructional model proposed by Trowbridge and Bybee [63]. Two mathematics experts were asked to examine the prepared lesson plans. The lesson plans were implemented after the revisions made following the expert opinions. A sample lesson plan is given below.

Engagement: The teacher asks the students what the word prism means. Then, the teacher distributes shapes containing different examples of prisms to the students and asks the students to examine these shapes and asks which shapes similar to these shapes they encounter in daily life.

**Figure 3:**
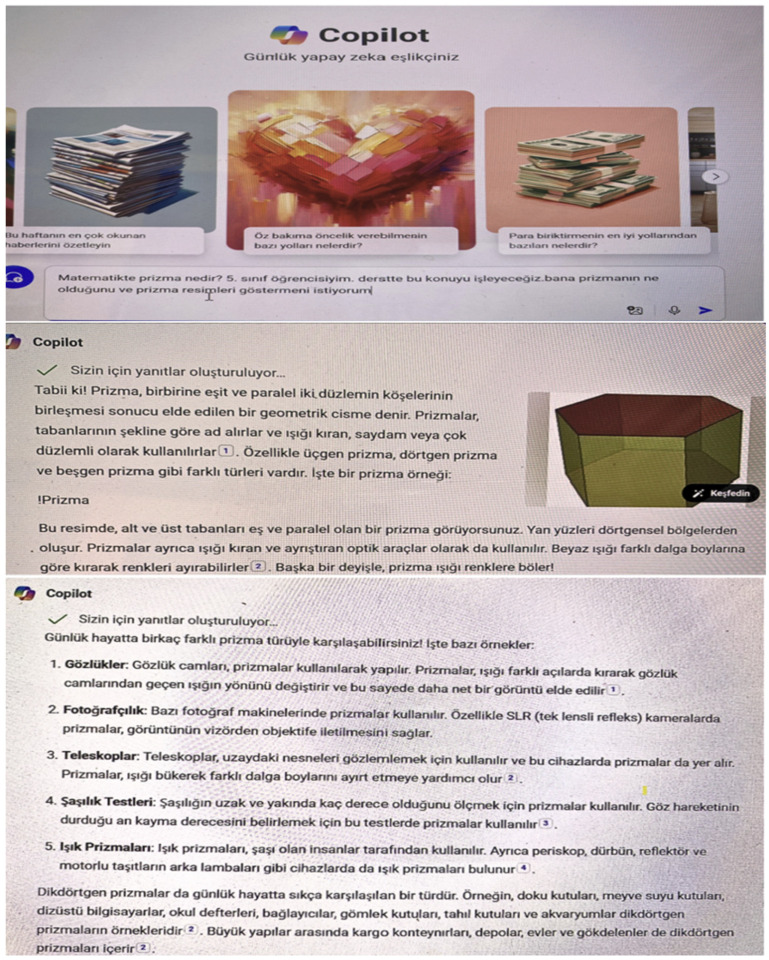
Co-pilot prism definition and examples student example.

**Figure 4:**
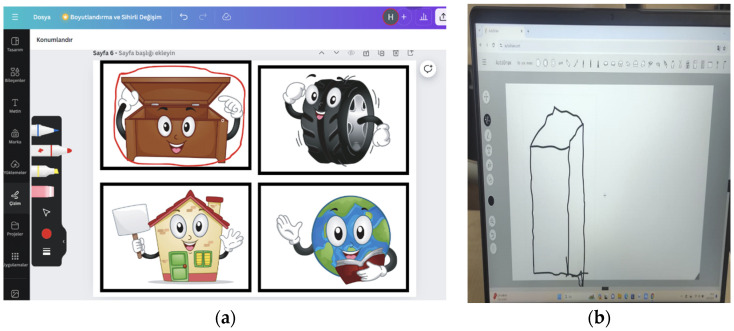
(**a**) Canva worksheet student example. (**b**) Autodraw prism drawing student example.

Explore: The Co-pilot (Microsoft 365 version) is asked to define the prism and present examples of prisms (see Figure 3). They are asked to circle the prism shapes from the worksheets prepared in Canva (Canva pro version) (see Figure 4a). They are asked to draw prism examples using the Quickdraw tool (see Figure 4b).

Explanation: The teacher gives the students the definition of a rectangular prism. Explains the basic elements of a rectangular prism. Gives the number of faces, vertices, and diagonals of a rectangular prism.

**Figure 5:**
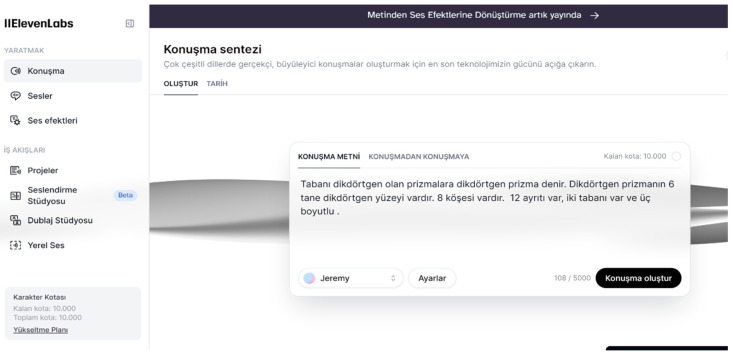
Example of a student voicing what they know about a rectangular prism with Eleven.labs.

Elaboration: Students are asked to vocalize what they have learned about the rectangular prism with the Eleven.labs (forever free version) tool in the classroom environment. Missing or incorrect parts are corrected together with the students, and in-depth learning is provided (see Figure 5).

Evaluation: The teacher uses a variety of assessment tools to evaluate the students’ learning process. Feedback is given to assess the understanding gained by the students during the lesson.

ChatGPT (ChatGPT 4o version) and Co-pilot (Microsoft 365 version) are used to obtain information about the subject and create stories during the teaching process in the experimental group; Auto-Draw (free version by Google Creative Lab) and Lexica.art (Lexica aperture v4) (drawing geometric objects quickly and accurately, seeing them from different angles, and realizing their esthetic aspects); Eleven.labs (forever free version), Fliki (free version), Suno.ai (Basic plan), and Animate from voice (free version) (exploring the properties of geometric objects in different emotional ways and increasing students’ interest in the subject); Geogebra Calculator 3D (5.2.850.0 version) (seeing and manipulating geometric objects in three dimensions) is used to see the expansions of prisms; worksheets prepared in Canva (Canva pro) are used for geometric objects; and DALL-3 (used Dall-e 3 ultra powered by ChatGPT) is used to create visuals for the story. The teacher guided the students during the use of the AI-supported tools and helped them when they misunderstood. The teacher’s role was to ensure that the lesson was conducted according to the lesson plan and to guide the students by asking questions. In addition, the results of the activities carried out using AI-supported tools were shared and discussed in the classroom environment. The teaching process in the experimental group is summarized as follows:

- AI activities were carried out in groups in the classroom.
- AI tools and worksheets were used: laptops, tablets, colored pencils, and blackboards.
- Working in groups of three (students constantly interacting with each other).
- Students reinforced what they learned with AI tools.
- Students were mostly active and guided by the teacher.

##### Sample Activity Prepared Using AI Tools

**Figure 6:**
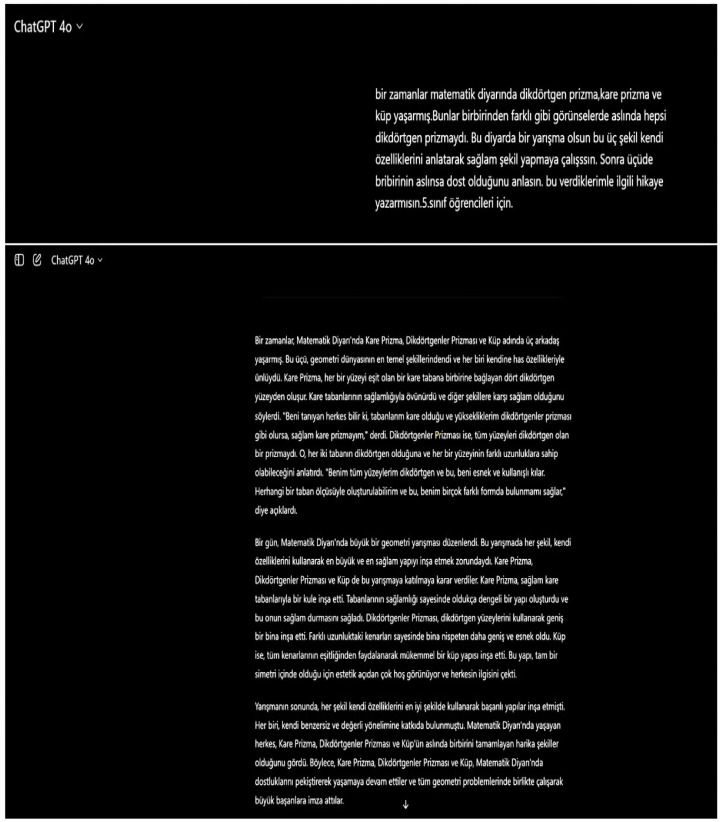
Example of creating a story by writing prompts with ChatGPT.

**Figure 7:**
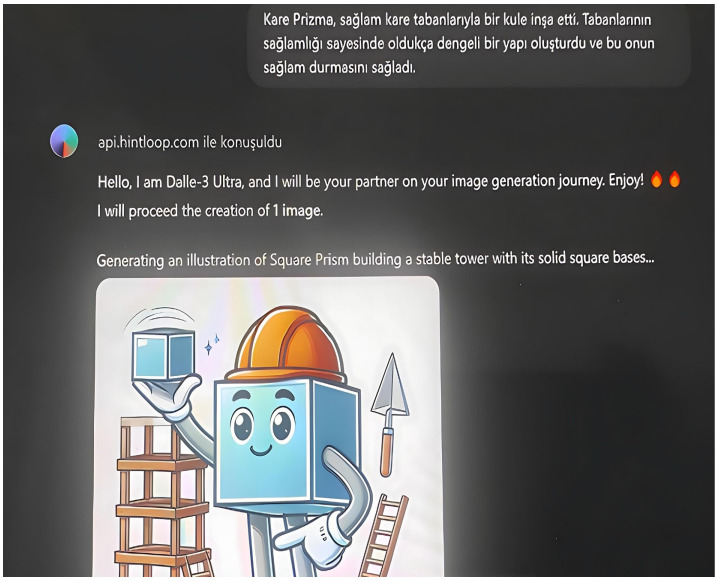
Example of creating a visual for a scene with DALL-3.

**Figure 8:**
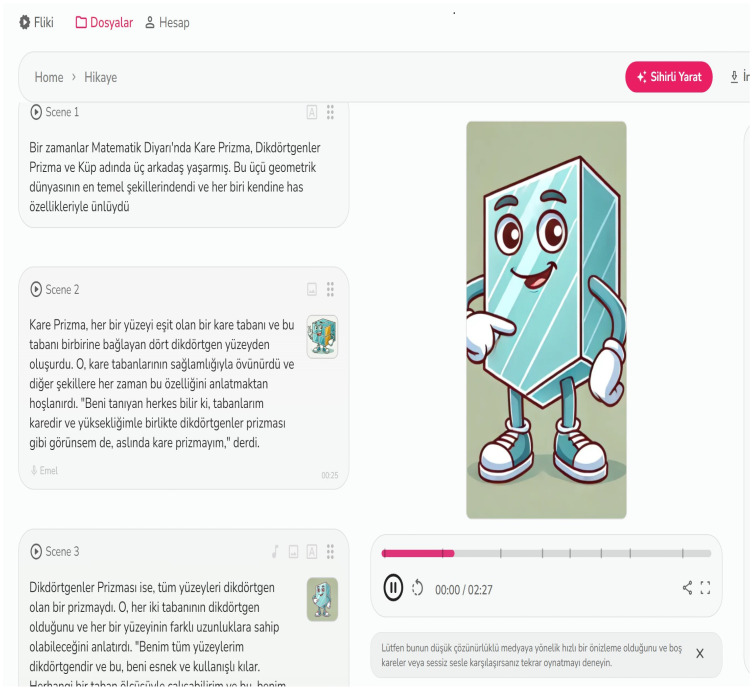
Example of digital story creation by combining scenes and images with Fliki.

**Figure 9:**
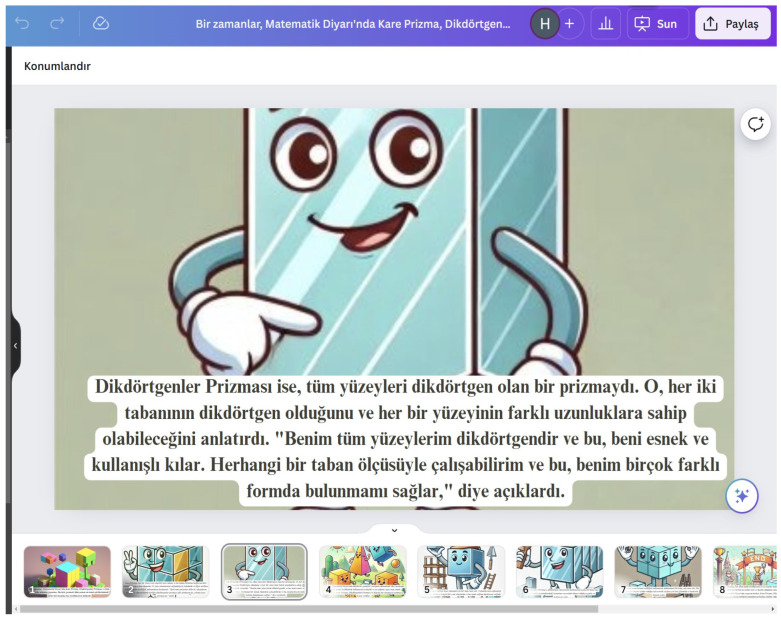
Student example of creating a story with visual content with Canva.

Using AI tools, stories about prisms were written and staged with visuals. In order to create a story, students first created a story by writing prompts in ChatGPT indicating what they wanted in their stories (see Figure 6). They divided the stories they created into scenes and asked the DALL-3 tool to create visuals for each scene (see Figure 7). They created a digital story by vocalizing each scene and the visuals of each scene with the Fliki AI tool (see Figure 8). They converted the digital story into a normal story using the Canva tool (see Figure 9).

#### 2.4.2. Control Group

In the control group, teaching took place in the students’ normal classrooms. The layout in the classroom was in the traditional seating style, with the students seated two to a desk. The lessons in the control group were taught by following the textbook, which is officially taught in the fifth grade. In this process, the teacher took part in the role of explaining and exemplifying the subject. The student remained passive in the lecture part and only answered the questions asked by the teacher. While the lessons were being taught, the activities in the textbooks at the end of the subjects were solved by the students in the classroom environment. In addition, in the problem-solving parts, interaction was provided between the individuals in the class, and the deficiencies or misunderstandings in the students were tried to be eliminated. In addition, at the end of each subject, the exercises in the book were given to the students as homework. The teaching process in the control group is summarized as follows:

- Teaching was carried out in a traditional classroom environment with desks one after the other.
- Activities from the curriculum textbook were used: blackboards, notebooks, and colored pencils.
- Students were individual (students did not interact much with each other).
- Students usually listened to the teacher and answered the teacher’s questions.

### 2.5. Data Analysis

The data were analyzed using IBM SPSS Statistics 23. The collected data underwent both descriptive and inferential statistical analyses. Descriptive statistics, including the mean and standard deviation, were utilized to summarize the data. For inferential statistics, analysis of covariance (ANCOVA) was employed to test the research hypotheses and determine whether there were significant differences between the control and experimental groups regarding the effect of the AI-supported 5E instructional model on the total scores of SVT, SRT, SOT, and SAT. In a pre-test–post-test control group design, if the researcher’s primary focus is on the effectiveness of the experimental procedure, the most appropriate statistical method is a single-factor ANCOVA with the pre-test scores controlled as a covariate [64]. The statistical significance of the differences between the pre-test and post-test scores of the experimental and control groups for SVT, SRT, SOT, and total SAT was interpreted at the 0.05 level. Before performing the ANCOVA, an independent samples t-test was used to examine whether the pre-test scores for SVT, SRT, SOT, and SAT total were equivalent between the experimental and control groups.

Before performing the ANCOVA test, the assumptions of the analysis—normality, homogeneity of variances, linearity, and homogeneity of regression slopes—were examined to ensure the validity and reliability of the statistical analysis. For normality, since the sample size was below 50, the Shapiro–Wilk test was used, and the skewness and kurtosis coefficients were also examined. The Shapiro–Wilk test considered *p* > 0.05 as indicative of normality, and for skewness and kurtosis, values between +2 and −2 were considered acceptable [65]. Levene’s test was used to assess the homogeneity of variances, with *p* > 0.05 indicating that the variances were homogeneous. For linearity, the correlation between the covariate and the dependent variable was examined to ensure a significant relationship. The equality of regression slopes was checked using the ANCOVA test, with slopes considered equal if *p* > 0.05. After confirming that all assumptions were met, the ANCOVA test was conducted to determine if there was a significant difference between the post-test scores of the groups. The effect size, measured by eta squared (η^2^), was calculated to determine the relative magnitude of this difference.

**Figure 10:**
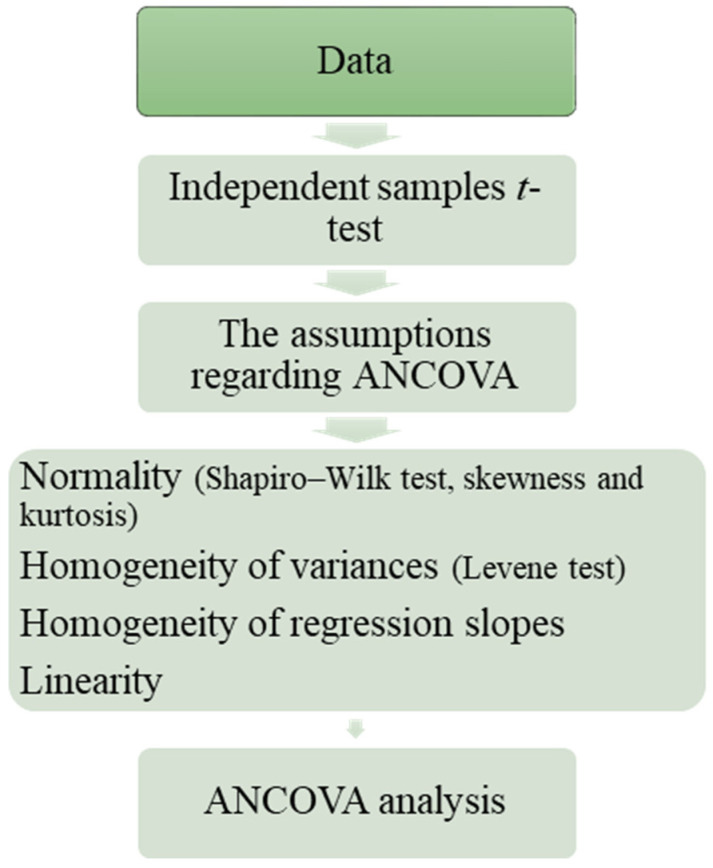
The diagram describing the data analysis process.

The diagram following the analysis of the data in this study was shown in Figure 10.

## 3. Results

### 3.1. Descriptive Statistics Results for Pre-Test and Post-Test Scores

Descriptive statistics results for the pre-test and post-test scores of the control and experimental groups for SAT and its sub-dimensions SVT, SRT, and SOT are presented in Table 1. This indicates that the SVT, SRT, SOT, and SAT total scores in the EG increased following the intervention.

**Figure 11:**
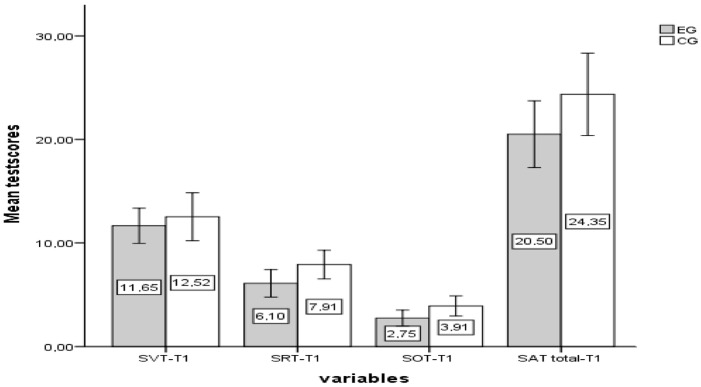
The pre-test (T1) results of experimental and control groups for SAT (sub-dimensions: SVT, SRT, and SOT).

Before the experimental procedure, an independent samples *t*-test was conducted to equate the pre-test scores of the groups. According to the test results, there was no significant difference between the pre-test scores of the experimental group (M = 24.35, SD = 9.20) and the control group (M = 20.50, SD = 6.89) for SAT; the experimental group (M = 12.52, SD = 5.35) and the control group (M = 11.65, SD = 3.63) for the SVT sub-dimension; the experimental group (M = 7.91, SD = 3.20) and the control group (M = 6.10, SD = 2.82) for the SRT sub-dimension; and the experimental group (M = 3.91, SD = 2.23) and the control group (M = 2.75, SD = 1.65) for the SOT sub-dimension. The test results were as follows: [*t*(41) = −1.53, *p* > 0.05; t(41) = −0.615, *p* > 0.05; *t*(41) = −1.95, *p* > 0.05; *t*(41) = −1.91, *p* > 0.05] (see Figure 11). These test results indicate that the control and experimental groups had similar prior knowledge before the experimental procedure.

### 3.2. Findings Related to SAT and Its Sub-Dimensions Post-Test Results of Experimental and Control Groups

In this study, a one-way analysis of covariance (ANCOVA) was used to compare the post-test means of the groups, with the pre-test scores considered as covariates. However, before performing the ANCOVA, it was necessary to check whether the study met the assumptions required for ANCOVA. Therefore, the study data were examined for normality, homogeneity of variances, homogeneity of regression slopes, and linearity.

The normality assumptions of the data were evaluated using the Shapiro–Wilk test, while the assumption of homogeneity of variances was assessed using the Levene test, and the results are presented in Table 1.

As indicated in Table 1, skewness and kurtosis values for all groups ranged between +2 and −2, with *p*-values greater than 0.05. According to the results of the Shapiro–Wilk test, both the pre- and post-test scores of the control and experimental groups followed a normal distribution. Therefore, it can be concluded that the normality assumption for the pre-test and post-test scores of the groups is met. Furthermore, the Levene test results in Table 1 indicated that the variances were homogeneous across groups (*p* > 0.05).

The assumptions regarding homogeneity and linearity of regression slopes are detailed in Table 2. To assess the homogeneity of the regression slopes, the interaction effect of the pre-test group on the post-test variables was examined. Additionally, to test for linearity, the relationship between the variables was evaluated.

Based on the analysis results presented in Table 2, it was found that the assumption of homogeneous regression slopes was supported for all variables (*p* > 0.05). Specifically, the common effect of pre-test group interaction on post-test scores was not statistically significant for SAT total [F(1, 41) = 1.895, *p* > 0.05]. Similarly, for the SAT sub-dimensions SVT, SRT, and SOT, the interaction effects of the pre-test group were also not significant [F(1, 41) = 0.635, *p* > 0.05; F(1, 41) = 0.319, *p* > 0.05; F(1, 41) = 0.029, *p* > 0.05], respectively. These findings indicate that there were no significant differences in the relationship between the covariate (pre-test scores) and the dependent variables (SAT sub-dimensions: SVT, SRT, and SOT) across the groups. Additionally, Table 2 shows a linear relationship between the pre-test and post-test total scores for SAT (SVT, SRT, and SOT) variables (*p* > 0.05). This confirms that the assumption of linearity between the covariate (pre-test scores) and the dependent variable (post-test scores) was met. In summary, the study’s data analysis supports that the assumptions of homogeneous regression slopes and linearity were upheld, ensuring the validity of the ANCOVA approach used to compare the groups’ post-test means while controlling for pre-test scores.

### 3.3. Findings Related to ANCOVA for SAT and Its Sub-Dimensions

Since all assumptions for ANCOVA have been satisfied, the analysis proceeded with ANCOVA to investigate whether there were significant differences between groups in SAT (SVT, SRT, and SOT) scores while controlling for pre-test scores.

**Figure 12:**
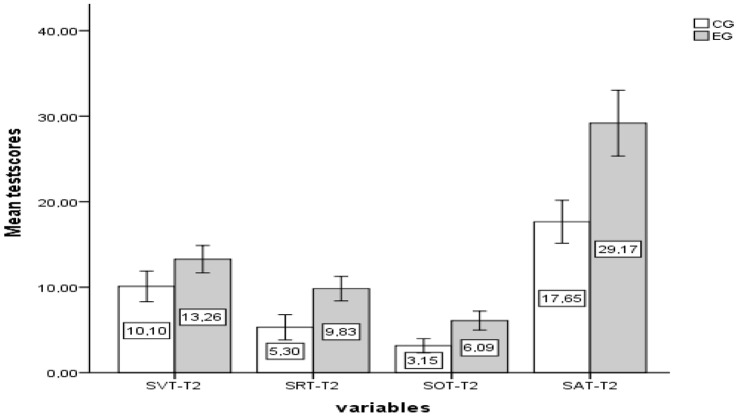
The post-test (T2) results of experimental and control groups for SAT (sub-dimensions: SVT, SRT, and SOT).

The ANCOVA analysis results, which assess the impact of AI-supported instruction on post-test scores while adjusting for pre-test scores in SAT and its sub-dimensions (SVT, SRT, SOT), are presented in Table 3 and Figure 12.

According to the ANCOVA results, after controlling for the pre-test scores of SVT, significant differences were found in the adjusted post-test scores between the experimental group (M = 13.03, SE = 0.56) and the control group (M = 10.36, SE = 0.61) for spatial visualization (F(1, 39) = 10.15, η^2^ = 0.20, *p* < 0.05). Bonferroni post hoc analysis indicated that this difference favored the experimental group significantly [mean difference = 2.66, *p* < 0.05]. The effect size, measured by partial eta squared (η^2^ = 0.20), suggests that 20% of the variability in SVT scores in the experimental group can be attributed to AI-supported teaching. According to Cohen [66], standards, this effect size is considered quite large. These findings indicate that using an AI-supported 5E instructional model had a significantly greater impact on fifth-grade students’ spatial visualization skills compared to the traditional textbook-based curriculum. According to the ANCOVA results, after controlling for the pre-test scores of SRT, significant differences were found in the adjusted post-test scores between the experimental group (M = 9.29, SE = 0.56) and the control group (M = 5.91, SE = 0.60) for understanding of spatial relationships (F(1, 40) = 16.06, η^2^ = 0.28, *p* < 0.05). Bonferroni post hoc analysis indicated that this difference favored the experimental group significantly [mean difference = 3.37, *p* < 0.05]. The effect size, measured by partial eta squared (η^2^ = 0.28), indicates that 28% of the variability in SRT scores in the experimental group can be attributed to AI-supported teaching. According to Cohen [66], guidelines, this effect size is considered quite large. These findings suggest that using an AI-supported 5E instructional model had a significantly greater impact on fifth-grade students’ understanding of spatial relationships compared to the traditional textbook-based curriculum. According to the ANCOVA results, after controlling for the pre-test scores of SOT, significant differences were observed in the adjusted post-test scores between the experimental group (M = 5.72, SE = 0.38) and the control group (M = 3.56, SE = 0.41) for spatial orientation (F(1, 40) = 13.90, η^2^ = 0.25, *p* < 0.05). Bonferroni post hoc analysis determined that this difference favored the experimental group significantly [mean difference = 2.15, *p* < 0.05]. The effect size, indicated by partial eta squared (η^2^ = 0.25), suggests that 25% of the variability in SOT scores in the experimental group can be attributed to AI-supported teaching. According to Cohen [66], this effect size is considered quite large. These findings demonstrate that using an AI-supported 5E instructional model had a significantly greater impact on fifth-grade students’ spatial orientation compared to the traditional textbook-based curriculum. According to the ANCOVA results, after controlling for the pre-test scores of SAT total, significant differences were observed in the adjusted post-test scores between the experimental group (M = 27.93, SE = 1.03) and the control group (M = 19.07, SE = 1.11) for overall SA (F(1, 40) = 32.957, η^2^ = 0.45, *p* < 0.05). Bonferroni post hoc analysis indicated that this difference favored the experimental group significantly [mean difference = 8.85, *p* < 0.05]. The effect size, measured by partial eta squared (η^2^ = 0.45), suggests that 45% of the variability in SAT scores in the experimental group can be attributed to AI-supported teaching. According to Cohen [66], this effect size is considered very large. These findings underscore that using an AI-supported 5E instructional model had a significantly greater impact on fifth-grade students’ spatial abilities compared to the traditional textbook-based curriculum.

## 4. Discussion

The primary aim of this study is to propose a learning environment enriched with AI-supported tools to improve the spatial abilities of middle school students. Sutton et al. [67] assert that the ability to extract 3D features from 2D representations is a fundamental aspect of SA, while Pittalis and Christou [17] emphasize the importance of 3D geometry instruction in enhancing middle school students’ spatial skills. Ben-Chaim, Lappan, and Houang [68], and Battista and Clements [69] indicate that SA can be improved through appropriate tools and activities. In our study, we aim to determine the contribution of students engaging with 3D objects using AI tools to their spatial abilities. Contero et al. [70] and Lohman [16] categorize SA into three dimensions: SVT (Spatial Visualization Test), SRT (Spatial Relations Test), and SOT (Spatial Orientation Test). This study also focuses on these three dimensions of SA.

This study has demonstrated that the AI-s5E instructional model has a significant and positive impact on students’ SAT skills. This finding is consistent with existing literature indicating that 3D geometry instruction enhances students’ spatial skills [17,71]. Furthermore, our study supports findings from research indicating the positive and significant effects of robot-based 3D applications on SAT scores [72,73]. Contero et al. [70] argue that applications such as creating computer-supported 3D models from freehand 2D drawings improve spatial ability, a conclusion supported by our study’s consistent results. Using appropriate software to animate shapes and mathematical representations obtained in AI enhances students’ imaginations [55]. For instance, software like DALL-E 3 and Lexica Art used in our study provided students with original realistic images based on their descriptions of 3D geometric shapes, enriching their visual knowledge of objects. This finding is consistent with the literature, as imagining objects mentally is crucial in SA [74]. Individuals with developed spatial abilities are often successful in various everyday interactions with our surroundings, such as using maps, recognizing and manipulating objects, planning routes, recalling locations, and creating art [75]. Studies [73,74,76,77] emphasize that developing SA supports success in fields like science, technology, mathematics, biology, chemistry, architecture, or engineering. Furthermore, research claims that SA is a significant predictor of mathematical success [78,79]. Clements [20] highlights the importance of SA in learning many topics in mathematics and geometry. In this context, it can be argued that by improving students’ spatial abilities, their mathematics achievement is also indirectly enhanced. This is because students with well-developed spatial abilities may be more successful in visualizing mathematical concepts and have stronger skills in solving geometric problems. Therefore, strengthening spatial abilities can increase students’ success in mathematics.

This study has demonstrated that the AI-s5E instructional model has a significant and positive impact on students’ SVT skills. This finding aligns with previous research highlighting the positive and significant effects of AI tools, particularly robot-based three-dimensional applications, on SVT [40,80,81,82]. Our study is consistent with Gao’s [83] finding that AI enables students to further develop their mathematical and cognitive skills while learning. AI tools, designed with human-like features or appearances, aim to support students in online learning environments [84]. As students today are more inclined to learn and explore new information independently, these powerful AI tools provide the opportunity to discover more desired knowledge without the need for assistance. Through these tools, students gain the ability to design their own products, which enhances learning as individuals design and construct their personal creations [85]. Clements [20] suggested that children’s spatial visualization abilities can be enhanced by working on creating and transforming images of two-dimensional and three-dimensional objects. AI tools offer skill-specific practice and provide reteaching and corrections when a student gives an incorrect answer, facilitating meaningful learning [86]. For instance, in our study, students used the Auto-Draw AI tool to create various prism drawings, and even amateurish prisms drawn by the students were transformed into more detailed professional drawings through the tool. Additionally, students were able to draw the nets of prisms using Geogebra Calculator 3D 5.2.850.0 software. This not only enhances students’ imagination but also aids in better understanding of the subject matter. This finding supports the results of Voskoglou and Salem [55], which indicate that AI software enhances individuals’ imagination. Therefore, these applications positively impact spatial visualization skills, which include the ability to perceive objects from different perspectives, mentally create and manipulate images of 2D and 3D objects [16,87], and imagine the movements of objects and spatial forms [88]. In this context, it can be argued that spatial visualization is a crucial skill for developing new ideas, designing new technologies, and comprehending complex data, which is particularly important for scientists and engineers. When students realize they can command these machines to perform desired tasks, they gain a sense of power over the technology, which can even increase their motivation towards the subject [89].

This study has demonstrated that the AI-s5E instructional model has a significant and positive impact on students’ SRT skills. This finding is consistent with studies on the role of robotics in enhancing students’ mental rotation skills [41,72,90]. Since AI enhances students’ imagination [55], it also contributes to the mental formation of concepts. Spatial relationship, which is the ability to mentally rotate an object quickly and accurately [17], is also supported by our study, aligning with these findings in the literature. Through AI tools, students have the opportunity to repeatedly view and modify different shapes and appearances of objects on the screen, which further supports their spatial relationships. Similarly, this study has demonstrated that the AI-s5E instructional model has a significant and positive impact on students’ SOT skills, one of the components of SA. This finding is consistent with studies on the role of robotics in enhancing students’ SOT skills [40]. Since AI enhances students’ imagination [55], it also contributes to the development of spatial orientation, which is the ability to visualize objects from different perspectives [91]. Spatial orientation can enable individuals to better perceive environmental elements and changes, which can help people make more effective decisions in daily life and various professional fields. Additionally, spatial orientation can improve individuals’ hand–eye coordination and motor skills—a critical skill for success in surgery, engineering, and various sports.

AI-based voice assistants can facilitate learning by enabling the creation of intelligent content [92] and engaging multiple senses. For example, in our study, AI-based voice assistants like Eleven Labs and applications such as Animate from Voice, which allow texts to be narrated by avatars, were used. Students were able to create content by adding voice to their written texts. This approach caters to both visual and auditory senses, addressing individual differences among students who learn in various ways, thereby facilitating learning for the entire class. Indeed, it has been suggested that AI offers a more personalized approach to meet the needs of the scientific community [93,94,95,96,97]. The findings of our study support this claim. Additionally, AI technologies analyze interactive content and learning behaviors, providing instant support or feedback to individual students [4,98,99,100,101,102]. In our study, students were asked to use the ChatGPT 4o software to create stories by inputting only a few commands. These stories provide information about what 3D geometric objects are and how they are used in daily life.

## 5. Conclusions

The primary aim of this study is to propose a learning environment enriched with AI-supported tools to improve the spatial abilities of middle school students. Because of it, the AI-s5E instructional model enhances the spatial abilities of fifth-grade students. Lesson plans were designed to enable students to explore new topics using AI tools, and these plans included practical sessions in which students engaged in hands-on learning. In this context, students were randomly divided into two groups: the experimental group, which participated in lessons using the AI-s5E instructional model, and the control group, which did not.

This study assessed the changes in spatial ability (SA), spatial visualization (SV), spatial relations (SR), and spatial orientation (SO) skills among students in the experimental group who participated in AI-based courses in comparison to students in the control group who did not receive these courses. To assess SAT, SVT, SRT, and SOT, both groups used tests developed by Dokumacı Sütçü [13], which have established validity and reliability, as pre- and post-tests. The scores obtained by the EG and CG groups in the pre- and post-tests were analyzed and comprehensively evaluated. The results indicated that the EG students who participated in AI-based lessons showed a significantly greater increase in their SAT, SVT, SRT, and SOT abilities compared to the CG students who did not participate in these lessons. It was also determined that the change in students’ post-test scores was largely due to the AI application. These findings demonstrate that the use of the AI-supported 5E instructional model has a significant impact on the development of spatial skills such as visualizing objects in the mind, recognizing objects from different perspectives, and manipulating objects in various ways, compared to the traditional curriculum taught with textbooks for fifth-grade students. AI applications, which have a positive and significant impact on spatial skills, can be integrated into teachers’ lessons and even included in curriculum programs. It is crucial to develop training and support programs for teachers to enable them to use AI-supported educational applications effectively. Programs should be designed to provide guidance on how teachers can integrate AI tools into their lessons effectively, and training sessions should be organized to support this objective. Learning such concepts through hands-on activities can contribute not only to academic achievement but also to students’ affective characteristics such as motivation, critical thinking, and self-efficacy. Future studies on the same or different topics can further determine the impact of AI-supported education on various affective characteristics of students. It is also essential to collect regular feedback from students to assess the effectiveness of AI-supported educational applications. Therefore, studies should be conducted to reveal students’ attitudes and experiences with these technologies, thereby contributing to the continuous improvement of these applications. Additionally, research can be undertaken to compare the effects of various AI tools and application methods, thus identifying which tools and methods are most effective.

## Figures and Tables

**Table 1:** Descriptive statistic results for pre-test and post-test scores.

			Experimental Group							Control Group							HVT
			N	M (SD)	Min	Max	Skew.	Kurto.	Shapiro–Wilk Tests (*p*)	N	M (SD)	Min	Max	Skew.	Kurto.	Shapiro–Wilk Tests (*p*)	Levene Statistic (*p*)
Pre-test	SAT	SVT	23	12.52 (5.35)	0	21	−0.30	−0.31	0.983 (*p* = 0.954)	20	11.65 (3.63)	7	18	0.27	−1.08	0.933 (*p* = 0.180)	0.183 (*p* = 0.671)
		SRT	23	7.91 (3.20)	2	14	−0.18	−0.590	0.954 (*p* = 0.357)	20	6.10 (2.82)	1	12	0.13	0.07	0.964 (*p* = 0.634)	0.876 (*p* = 0.355)
		SOT	23	3.91 (2.23)	0	9	0.60	0.24	0.945 (*p* = 0.233)	20	2.75 (1.65)	0	6	0.44	−0.77	0.910 (*p* = 0.063)	0.485 (*p* = 0.490)
		SAT Total	23	24.35 (9.20)	5	38	−0.18	−0.88	0.952 (*p* = 0.329)	20	20.50 (6.89)	10	33	0.41	−0.90	0.943 (*p* = 0.270)	2.236 (*p* = 0.143)
Post-test	SAT	SVT	23	13.26 (3.70)	8	21	0.05	0.38	0.983 (*p* = 0.954)	20	10.10 (3.83)	5	19	0.79	0.07	0.933 (*p* = 0.180)	0.183 (*p* = 0.671)
		SRT	23	9.83 (3.29)	4	15	0.04	−0.69	0.945 (*p* = 0.228)	20	5.30 (3.16)	1	13	1.01	0.69	0.911 (*p* = 0.068)	0.279 (*p* = 0.600)
		SOT	23	6.13 (2.49)	2	10	−0.10	−0.80	0.914 (*p* = 0.051)	20	3.15 (1.78)	1	6	0.42	−1.20	0.883 (*p* = 0.020)	1.826 (*p* = 0.184)
		SAT Total	23	29.17 (8.89)	11	45	0.05	−0.44	0.977 (*p* = 0.858)	20	17.65 (5.38)	9	26	0.14	−0.82	0.940 (*p* = 0.239)	3.964 (*p* = 0.053)

Skew.: skewness; Kurto.: kurtosis; HVT: homogeneity of variances test.

**Table 2:** Results of the assumptions regarding homogeneity and linearity of regression slopes.

	Reception of Homogenous Regression Slope			Correlation Pre-Test and Post-Test		
Intervention	Variable	F	*p*	Variable	r	*p*
Al-s5E instructional model	SVT*Group	0.635	0.430	SVT	0.649	0.000
	SRT*Group	0.319	0.576	SRT	0.632	0.000
	SOT*Group	0.029	0.865	SOT	0.637	0.000
	SAT Total*Group	1.895	0.177	SAT	0.726	0.000

**Table 3:** Results of ANCOVA for post-test of AT.

Variable	Group	M	SD	Adjusted Mean	SE	F	Partial eta Squared (η^2^)
SVT	Experiment 1	13.26	3.70	13.03	0.59	10.15 *	0.202
	Control	10.10	3.83	10.36	0.62		
SRT	Experiment 1	9.83	3.29	9.29	0.56	16.06 *	0.286
	Control	5.30	3.16	5.91	0.60		
SOT	Experiment 1	6.13	2.49	5.72	0.38	13.90 *	0.258
	Control	3.15	1.78	3.56	0.41		
SAT total	Experiment 1	29.17	8.89	27.93	1.03	32.94 *	0.452
	Control	17.65	5.38	19.07	1.11		

* *p* < 0.05.

## Data Availability

The data will be shared upon a reasonable request from the corresponding authors.
